# A Monolithic Multisensor Microchip with Complete On-Chip RF Front-End

**DOI:** 10.3390/s18010110

**Published:** 2018-01-02

**Authors:** Massimo Merenda, Corrado Felini, Francesco G. Della Corte

**Affiliations:** 1 Department of Information Engineering, Infrastructure and Sustainable Energy (DIIES), “Mediterranea” University, Via Graziella Loc. Feo di Vito, 89124 Reggio Calabria, Italy; corrado.felini@unirc.it (C.F.); francesco.dellacorte@unirc.it (F.G.D.C.); 2 HWA srl-Spin Off dell’Università Mediterranea di Reggio Calabria, Via Reggio Campi II tr. 135, 89126 Reggio Calabria, Italy

**Keywords:** CMOS sensors, On-Chip-Antenna, LC oscillator, temperature sensor, light sensor, Internet of Things

## Abstract

In this paper, a new wireless sensor, designed for a 0.35 µm CMOS technology, is presented. The microchip was designed to be placed on an object for the continuous remote monitoring of its temperature and illumination state. The temperature sensor is based on the temperature dependence of the I-V characteristics of bipolar transistors available in CMOS technology, while the illumination sensor is an integrated p-n junction photodiode. An on-chip 2.5 GHz transmitter, coupled to a mm-sized dipole radiating element fabricated on the same microchip and made in the top metal layer of the same die, sends the collected data wirelessly to a radio receiver using an On-Off Keying (OOK) modulation pattern.

## 1. Introduction

Microchip sensors have the advantage that the transducers can be monolithically integrated with signal amplification and conditioning circuitry, with clear advantages in terms of reduced size, noise immunity and production costs. With the advent of the Internet-of-Things [1], these devices are expected to gain in popularity, as they can be considered the link between the world surrounding an object they are attached to and electronics. Of course, sensors that are readily deployable and can be easily accessed, possibly in a wireless manner, are highly desirable [2,3]. Nevertheless, the application of an external antenna to a microchip to allow wireless communication is a costly process and moreover leads to the de-miniaturization of the device [4]. Therefore, the concept of an on-chip-antenna (OCA), fabricated during the CMOS process, which eliminates the need for external transmission lines and sophisticated packaging, seems to be the simplest approach to warrant short range data transmission together with low costs and reliability. Usually, this task is obtained by integrating spiral inductors as the radiating elements [5,6,7,8]. In this paper, we present a full custom microchip integrating a dipole RF radiator fed by an LC oscillator and an RF power amplifier. The choice of a dipole antenna allowed the design of almost one-dimensional form factor microchips, while loop antennas require square devices. The device includes a temperature sensor, a light sensor and a general-purpose input and was designed for ambient and processes monitoring when temperature, illumination or other output signals need to be kept under control. Two examples of possible application scopes are photovoltaic cells in solar modules and light emitting diodes. By means of the integrated 2.5 GHz transmitter, the device wirelessly sends the collected data to a base station, placed up to a few meters away. The microchip was fabricated by a silicon foundry (Austria Micro Systems), with a standard and low cost 0.35 μm CMOS process (C35B4M3) [9], which demonstrates that even low cost technologies are suitable for this kind of fully integrated sensors.

The paper is organized as follows. In Section 2 we present the microchip architecture with a general description of its internal organization. In Section 3 we provide details about the integrated sensors, the analogue front-end, the control logic and the RF section. In Section 4 we provide experimental results on the microchip operation. Finally, the conclusions are drawn in Section 5.

## 2. System Architecture 

**Figure 1:**
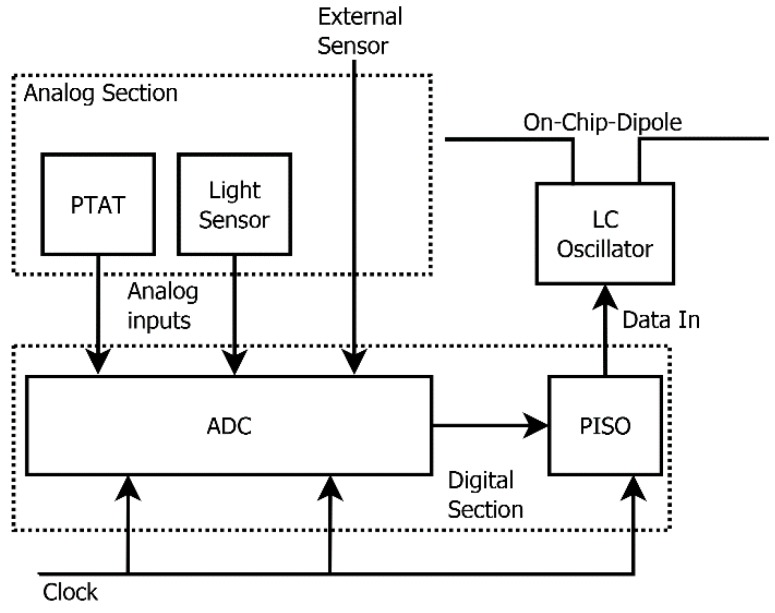
Wireless multisensor architecture.

The architecture of the proposed device is shown in Figure 1. The analogue section includes two sensors, namely a temperature sensor and a light sensor. The former is designed to provide a “proportional to absolute temperature” (PTAT) output and guarantees an optimal linearity; the latter is in fact an integrated solar cell, designed to provide a useful output under normal outdoor daylight irradiation. Sensor outputs are first amplified to bring their respective signals to useful levels and then digitalized by means of a single analogue-to-digital converter (ADC). The ADC is time-shared between either two or three sources, namely the PTAT, the light sensor and in case a generic input applied between two input pads of the microchip, where an additional external sensor can be applied. The serial conversion timing is provided by the digital section. 

The ADC serial outputs are afterwards loaded into a parallel-in/serial-output (PISO) 2 × 8 (or 3 × 8) bits shift register. Once the measurements are done and the PISO loaded, its content is serialized for transmission at a bit-rate of 3 kbps. The microchip is also internally provided with an integrated RF transmitter, composed of an LC-oscillator, turned on and off by the bitstream and an RF power amplifier (PA), in turn energizing an integrated small dipole antenna.

## 3. Microchip Blocks Description

### 3.1. Temperature Sensor

Low cost high-performance temperature sensors are increasingly required. Besides, it is needed to reduce their power consumption. This concept is necessary to use the sensor in battery operated systems, to decrease the errors produced by self-heating and to be able to add the sensor to a chip without causing a significant increase in the power consumption of the complete system [10].

An integrated CMOS temperature sensor perfectly fits low cost, high performance and low power consumption.

Several temperature sensors topologies have been proposed in CMOS technology. Most of the systems reported in literature, including the one presented hereafter, exploit a PTAT scheme, realized by means of coupled bipolar junction transistors (BJT) [11,12].

In fact, assuming for PNP BJTs the collector current given by:

(1) $$I_{C}=I_{S}e^{\left(\frac{qV_{EB}}{\eta kT}\right)},$$

with *I_s_* the saturation current of the emitter-base junction, *V_EB_* the emitter-base voltage, *k* the Boltzmann constant, *q* the electronic charge and η the ideality factor, if two identical transistors $\left(I_{S1}=I_{S2}=I_{S}\right)$ are biased at collector currents of $I_{ref}$ and Io respectively, the difference between the emitter-base voltages of the two BJTs has a linear dependence on *T*, according to the following equation.

(2) $$\Delta V_{EB}=V_{EB1}-V_{EB2}=V_{t}\ln\frac{I_{ref}}{I_{s}}-V_{t}\ln\frac{Io}{I_{s}}=\frac{kT}{q}\ln\left(\frac{I_{ref}}{Io}\right),$$

where η=1 is assumed.

Our sensor was designed to provide a high sensitivity and a signal level tuned in order to simplify the Analog-to-Digital conversion (ADC) in the temperature range from room temperature to 100 °C.

**Figure 2:**
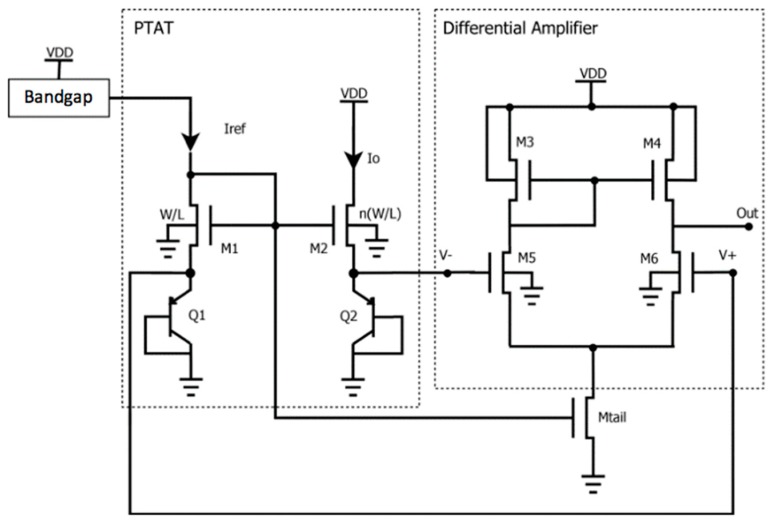
Architecture of the integrated PTAT with differential amplifier.

The temperature sensor consists of two building blocks: a PTAT voltage generator and a differential amplifier, as shown in Figure 2.

To obtain a proportional output signal with respect to absolute temperature, two diode-connected BJTs, driven by different currents, were realized. The two PNP transistors Q1 and Q2, both working in the active region, are realized side-by-side on the chip and have the same geometry and orientation and therefore can be assumed identical. The ratio between their collector currents is fixed by the different W/L aspect ratios of M1 and M2. Assuming in principle negligible channel length modulation effects [13], Q2 is driven by a current *Io* that is $n\times I_{ref}$, where *n* is the channel width ratio W_M1_/W_M2_ while L_M1_ = L_M2_. In this work *n*=10 was chosen for an appropriate sizing of the MOS transistor as trade-off between sensitivity in the measured temperature range and linearity of the output signal.

The reference current $I_{ref}$ is generated through an integrated Bandgap current reference [14] with supply and temperature compensation that delivers a constant current of 11.5 μA, showing a temperature dependency of about 800 ppm/°C. As shown by the simulations, this temperature dependency has no practical impact on the performance of the two sensors. The source is designed to supply each diode-connected BJT with currents that are independent from $V_{dd}$. 

**Figure 3:**
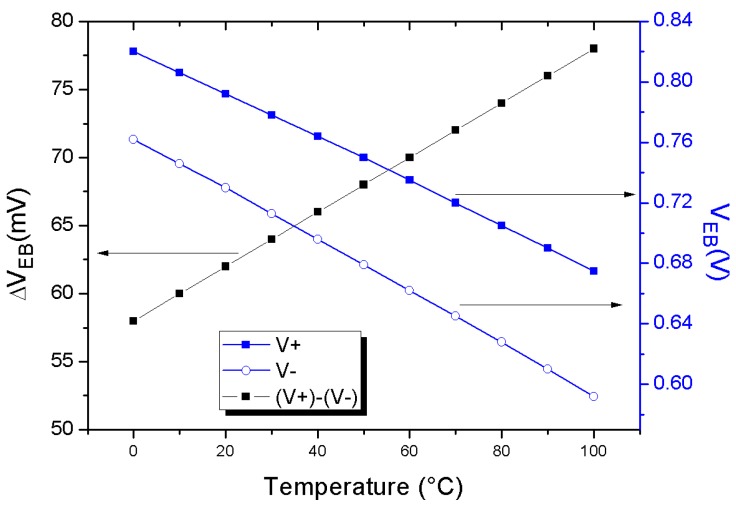
Emitter-base voltage of transistors Q1 and Q2 at various temperatures (**right scale**) and differential output of the PTAT (**left scale**) in the temperature range from 0 to 100 °C. The lines are guides to the eye.

In Figure 3 the simulated emitter-base voltages of transistors Q1 and Q2 at various temperatures are shown. Their behaviour shows a negative derivative with respect to temperature. From simulations, it is possible to calculate the sensor output at each absolute temperature:

(3) $$V_{PTAT}\left(T\right)=V_{+}-V_{-},$$

In the same Figure, the differential output of the PTAT in the temperature range from 0 to 100 °C is shown.

In the temperature range of interest, the estimated power consumption of the two PNP transistors is 7.7 μW and 66.5 μW respectively for Q1 and Q2, obtained from simulations. This power might cause self-heating of the sensor and therefore the PTAT circuit is activated only for a few milliseconds through the Bandgap circuit at regular intervals.

The amplifier stage was designed through extensive parametric simulations aimed at obtaining output signal levels that could be reasonably applied to the ADC, while preserving a good linearity. Here, M3 and M4 are used as active load, instead of using high value resistors, whose implementation is wasteful in terms of area consumption. M5 and M6 have been sized to achieve the maximum output dynamics for the differential amplifier. Being the PTAT output differential, the topology requested for the amplifier is differential as well. M_tail_ has the role of a current limiter for the amplifier, in order to reduce power consumption.

### 3.2. Light Sensor

**Figure 4:**
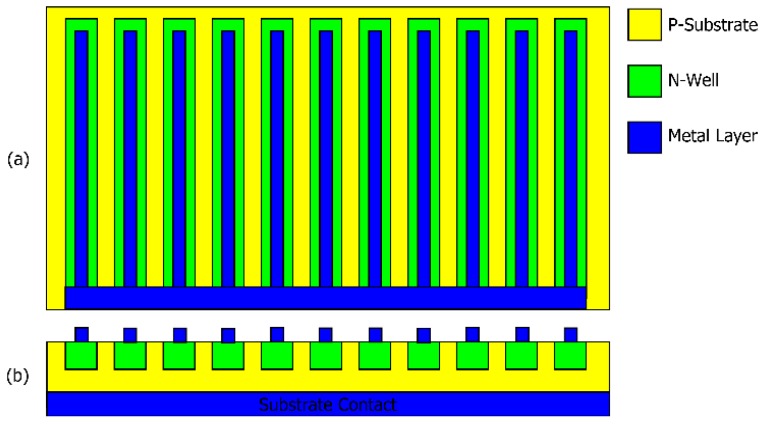
Structure of the realized photodetector: (**a**) top view; (**b**) cross section (figure not in scale).

The proposed solution for the design of the CMOS light sensor is based on planar p-n junction formed by the P-type substrate and the N-type well. This structure could be figured out as a micro solar cell producing a current proportional to surface irradiation. The outermost metal layer (Metal-1 for the CMOS technology) was used to build fingers that collect photocurrent at the cathode. The structure of the light sensor is shown in Figure 4.

**Figure 5:**
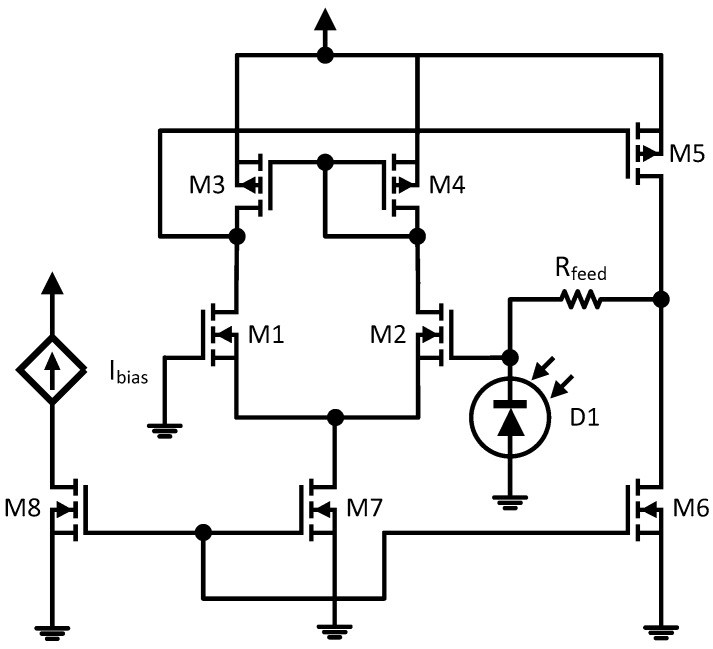
Integrated light sensor with transimpedance amplifier.

In order to convert the current signal into a voltage signal, it is necessary to use a transimpedance amplifier (TIA), designed according to the schematic depicted in Figure 5.

**Figure 6:**
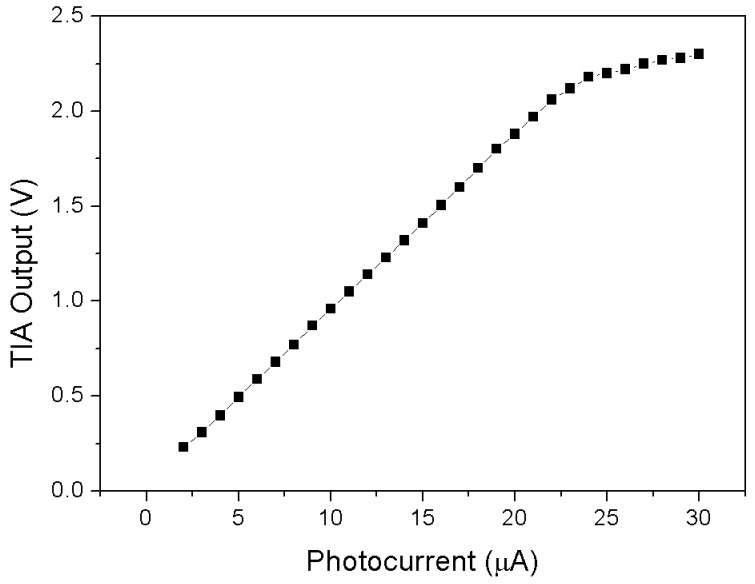
Simulated characteristic of the transimpedance amplifier output vs photocurrent.

The integrated solar cell area (300 × 200 μm^2^) was chosen to generate a photocurrent in a range that suits the linear region of the amplifier (up to 22 μA). By assuming an indicative maximum efficiency of 10%, the area was chosen in such a way that under 1000 W/m^2^ the cell would still provide a photocurrent within the said limits. The TIA is based on an integrated MOSFET operational amplifier. This operational amplifier performs an open loop gain of 100 dB with an output resistance of 0.3 Ω and can operate in the temperature range from −40 °C to 120 °C. The simulated TIA output is shown in Figure 6. The feedback resistor R_feed_ value is 100 kΩ.

### 3.3. Clock 

Conventional current-starved single-ended ring oscillators are usually employed for realizing wide tuning range, voltage controlled oscillators that act as local clock for the digital circuitry of ASICs by exploiting the dependence of the large signal oscillation frequency *f_Osc_* on the bias current *I_b_* [15,16] that is determined by:

(4) $$f_{Osc}=\frac{1}{2Nt_{D}}\approx \frac{I_{b}}{2NOscC},$$

where *N* is the number of inverters used, *t_D_* the time delay of each stage, *V_Osc_* the oscillation amplitude and C the load capacitance.

Furthermore, in a ring oscillator topology the small-signal amplitude of the oscillating signal can be expressed as

(5) $$V_{Out}\left(t\right)=v_{Out}\left(0\right)\exp\left(\frac{\left|A_{s}\right|-2}{2}\omega_{s}t\right)\cos\left(\frac{\left|A_{s}\right|\sqrt{3}}{2}\omega_{s}t\right),$$

where *v_Out_*(0) is the initial condition at the ring oscillator output, *A_s_* the gain of each amplifier stage and *ω_s_* the −3 dB bandwidth of each stage [13]. The first term of (5) represents the time-varying amplitude of the oscillations. Therefore, the settling time can be expressed to a first approximation as

(6) $$t_{s}=\frac{2}{\left(\left|A_{s}\right|-2\right)\omega_{s}}\ln\left(\frac{0.9V_{Osc}}{v_{Out}\left(0\right)}\right),$$

The settling time of a ring oscillator (RO), at a given initial condition, depends only on the amplifier stages characteristics (gain and bandwidth). In particular, when the oscillator is implemented by exploiting CMOS inverters, the small-signal gain of a single stage is given by

(7) $$A_{s}=-\left(g_{m_{n}}+g_{m_{p}}\right)r_{o_{n}}\|r_{o_{p}}$$

where *r_on_* and *r_op_* are the output resistances of the MOS devices. However, an increase of the transconductance of the inverting stages does not necessarily imply a reduction of the oscillator settling time because a higher gain requires more current consumption and thus larger size components with increased parasitics that tend to decrease *ω_s_*. To obtain a fast start-up oscillator, required for low power clocks, bias current and hence devices sizes have to be properly set. 

In this work, the oscillator provides a stable carrier of 10 MHz frequency (*W_p_*/*W_n_* = 4, *C*_1_ = *C*_2_ = *C*_3_ = 790 pF). For achieving this purpose, the same bandgap current reference introduced in Section 3.1 has been used, providing high robustness against supply voltage and temperature fluctuations. A clock signal of 5 kHz is thus obtained using a frequency divider (*f_in_*/*f_out_* = 2000). 

### 3.4. Digital Section

**Figure 7:**
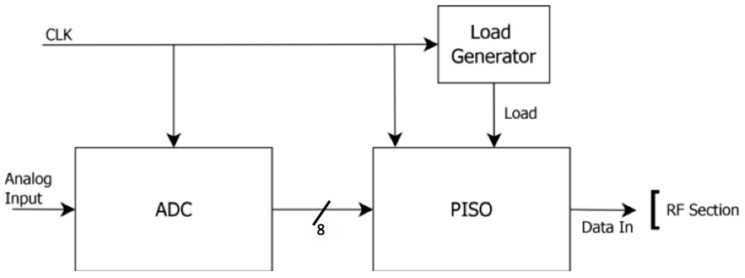
Block diagram of the Digital Section.

In Figure 7 the blocks of the digital section are shown. An ADC was used to convert the analogue signals produced by the sensors; a parallel-in-serial-output (PISO) shift register was implemented to serialize the parallel output of the ADC; a Load signal is necessary to activate the loading by the PISO. In this work, an integrated 8-bit successive approximation ADC was used, available in the standard cell library of the AMS technology.

**Figure 8:**
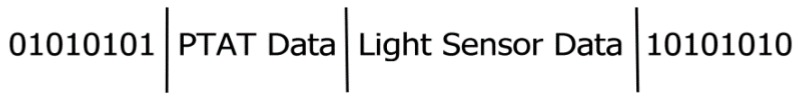
Serialized output of 32-bits PISO.

The PISO output normally consists of 4 × 8-bit words. Five words are used however when the external sensor is also selected. The first and the last words are used to identify Start and Stop of modulation. They can additionally contain a microchip read-only identifier. The entire sequence of bits is depicted in Figure 8. Between the first and the last 8-bits, there are the digital values of the PTAT and light sensor (payload).

The Load command generator was designed to generate a high-level every 50 cycles of the 5 kHz Clock.

### 3.5. RF Section

**Figure 9:**
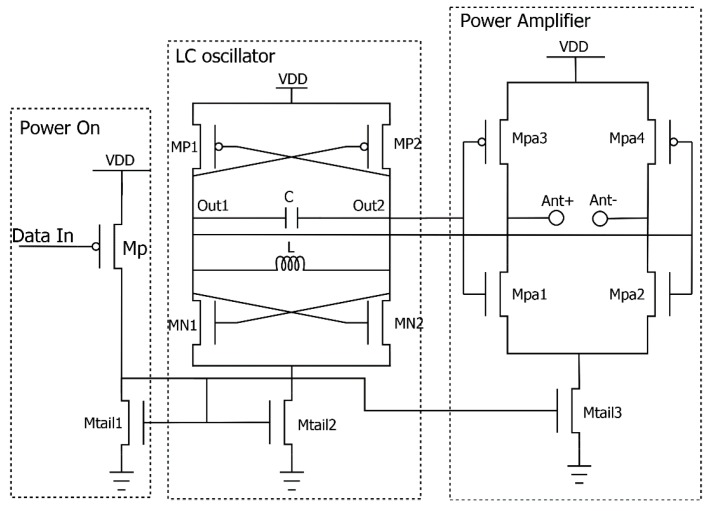
Circuit schematic of the implemented transmitter.

In this microchip sensor, a directly On-Off-Keying (OOK) modulated oscillator-based transmitter was used, as depicted in Figure 9. The transmitter topology exploits a cross-coupled complementary LC oscillator running at a frequency of 2.5 GHz. The differential output is coupled to a common source differential power amplifier and buffered on an integrated dipole antenna. A start-up circuit allows the duty cycling of the transmitter and the implementation of the OOK modulation.

LC cross-coupled oscillators are suitable circuits to be used in radio frequency systems. These kinds of oscillators offer high frequency stability over temperature, voltage and process tolerances and low phase noise [13]. 

In this oscillator topology, the oscillation frequency is set by an inductors-capacitors (LC) network according to the formula:

(8) $$f=\frac{1}{2\pi \sqrt{\mathrm{LC}}},$$

Despite the simplicity of the cross-coupled differential scheme, the complementary architecture features a higher negative transconductance useful for overcoming the resonating circuit losses, essentially due to the parasitic resistance of the LC tank and sustaining oscillations [17]. In particular the following relationship must be verified [18]:

(9) $$\frac{2}{G_{m}}\geq R_{tank},$$

where $G_{m}$ is the transconductance provided by the differential couple and $R_{tank}$ is the parasitic resistance of the LC tank.

The complementary topology allows oscillations to start also for low quality factors ($Q_{tank}$) of the resonating circuits, which is typical for fully integrated oscillators. In fact, the inductor shows a poor quality factor $Q_{L}$, typically in a range 4–20 for enhanced structures [19,20].

The resonating circuit employs L = 4 nH and C = 1 pF. In particular, we used 4-turns square spiral inductors fabricated in the top aluminium metal layer and polysilicon capacitors. The employed coils exhibit a quality factor of 4.7 at 2.5 GHz [9]. 

Each transistor has a W/L ratio set to achieve the needed negative transconductance for sustaining oscillations. Furthermore, the sizes of the cross-coupled pairs, together with the current tail, were chosen to properly bias the differential power amplifier. Table 1 summarizes the key dimensions of each transistor.

A linear power amplifier (PA) based on a class B bridge configuration amplification stage was implemented to feed the small dipole antenna. The differential MOSFET pairs were accurately sized for maximizing the power delivered to the antenna alternatively through the Mpa3-Mpa2 and Mpa4-Mpa1 paths. A W/L ratio of 65 μm/0.35 μm was set for all Mpa transistors. The estimated power gain is 2.3 dB with a total harmonic distortion (THD) of 0.13%, estimated from simulations. This stage is turned on by Mtail3 only during transmission.

The on-chip dipole radiating element is made within the top aluminium metal layer, having a thickness of 0.925 µm. This antenna requires a differential excitation, which suits the PA scheme. 

For a resonating antenna, the linear dimensions must be of the order of one half of the carrier wavelength. Therefore, for operations at 2.4 GHz, a well-tuned dipole antenna should have dimensions of the order of centimetres. However, the chip size limits the antenna dimension, so it is not possible even approaching the resonating conditions in our case. The characteristic parameters of such a small antenna are obviously very poor, with an estimated gain of about −50 dB and a radiation efficiency of the order of 1% [21], enough however to allow short range transmissions [6].

## 4. Experimental Results

The circuital sections were first separately characterized. In particular, tests were made on the analogue sensors (temperature and light), the digital conversion section with data serialization and finally on the RF transmitter. 

**Figure 10:**
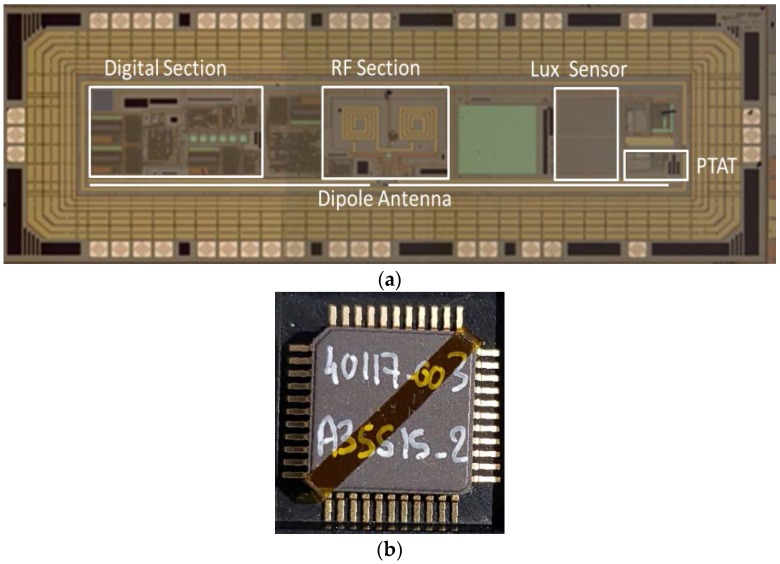
(**a**) Microphotograph of the realized chip. The main sections of the microchip are highlighted; (**b**) Microphotograph of an encapsulated microchip.

A microphotograph of the die is shown in Figure 10a. The area of the realized chip is 1 mm × 4 mm. After fabrication, for a complete characterization, the test chips were packaged in 44-pin TQFP packages (Figure 10b) which give easy access to all input and output nodes of the circuit. Bare dies samples were also tested after ultrasonic wire bonding to a limited number of bondpads, e.g., for RF transmission tests.

### 4.1. Temperature Sensor

**Figure 11:**
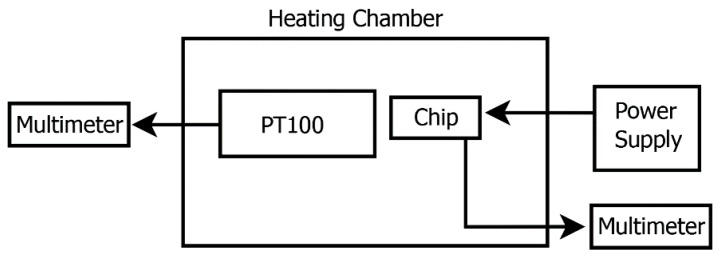
Experimental setup used to characterize the PTAT performances.

**Figure 12:**
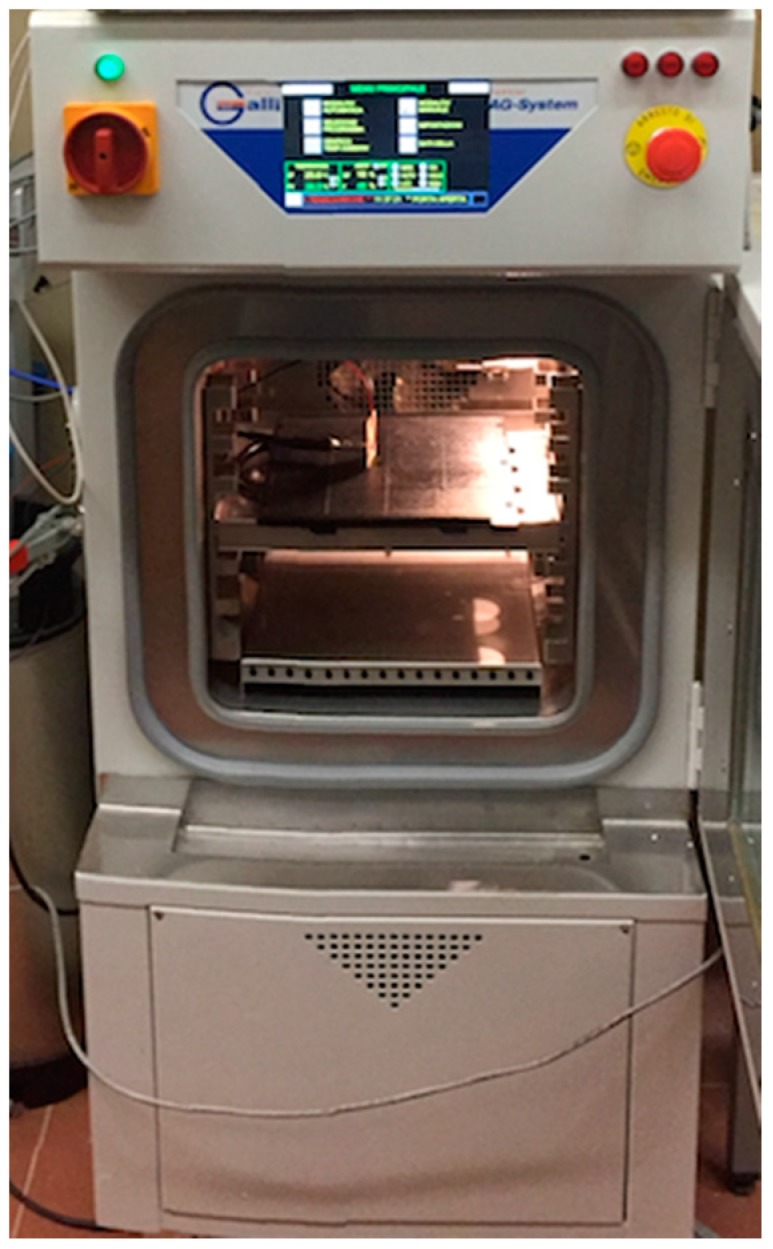
Picture of the setup used for the characterization of the temperature sensor.

To investigate the performance of the PTAT sensor, the whole chip was tested in a thermostatic oven, using a standard PT100 as reference as shown in Figure 11 and Figure 12.

**Figure 13:**
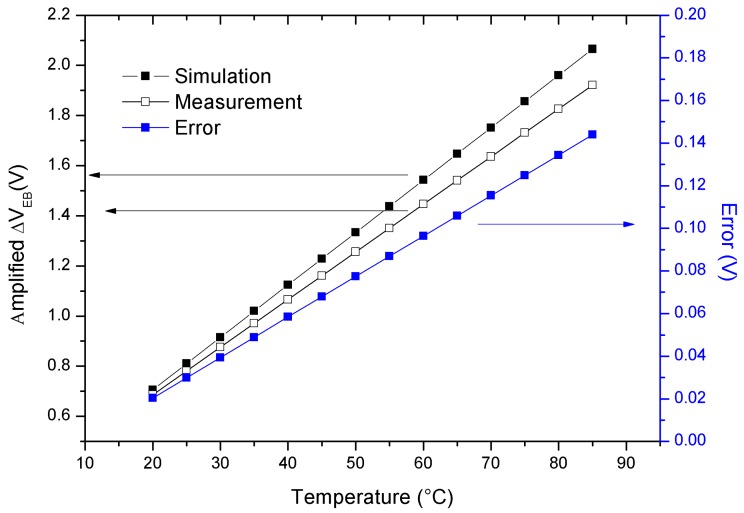
Experimental characteristic of the PTAT sensor, obtained using analogue output. The simulated characteristic is also provided for comparison. The error between simulated and experimental characteristic is also added.

As shown in Figure 13, the integrated PTAT presents a good linearity (*R*^2^ = 0.99962) in the temperature range of interest (20 °C–90 °C) with a sensitivity of 19.1 mV/°C, measured at the analogue amplifier output. Inside the climatic chamber it was possible to change in a small range the lighting conditions in combination with temperature. In particular, a modification of the LED array output, from 10 to 50 W/m^2^ approximately, was applied showing no effect at all on the temperature sensor output. However, it can be expected that higher irradiations would induce heating of the device.

This specific implementation of the sensor does not include a calibration circuitry, which could be however obtained in a final version by e.g., laser trimming acting on I_ref_ or other standard techniques.

Table 2 summarizes the PTAT key parameters.

### 4.2. Light Sensor

To examine the performance of the light sensor, the whole chip was exposed to direct sunlight and the photocurrent was measured at several irradiations (IRR), continuously monitored with a hand-held solar power meter. Photocurrent has been measured using an Agilent Technologies 4155C Semiconductor parameter analyser.

**Figure 14:**
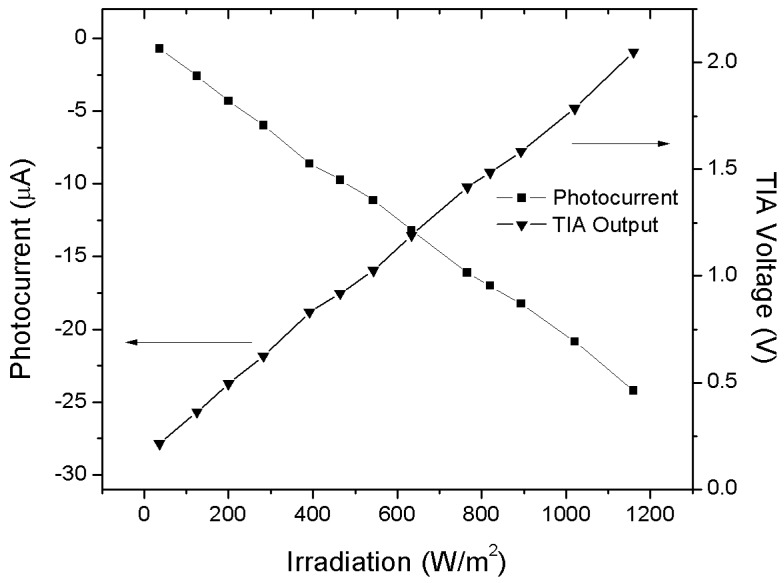
Characteristic of the irradiation sensor, obtained using analogue output.

As shown in Figure 14 the light sensor presents a good linearity (*R*^2^ = 0.99915) in the range of interest of a typical sunny day. The sensitivity is calculated as follows:

(10) SCel+Tia=ΔVTIAΔIRR=16.96 mVW/m2,

In this specific implementation of the sensor, an additional calibration feature was not considered, though it would be possible by e.g., laser trimming of R_feed_.

**Figure 15:**
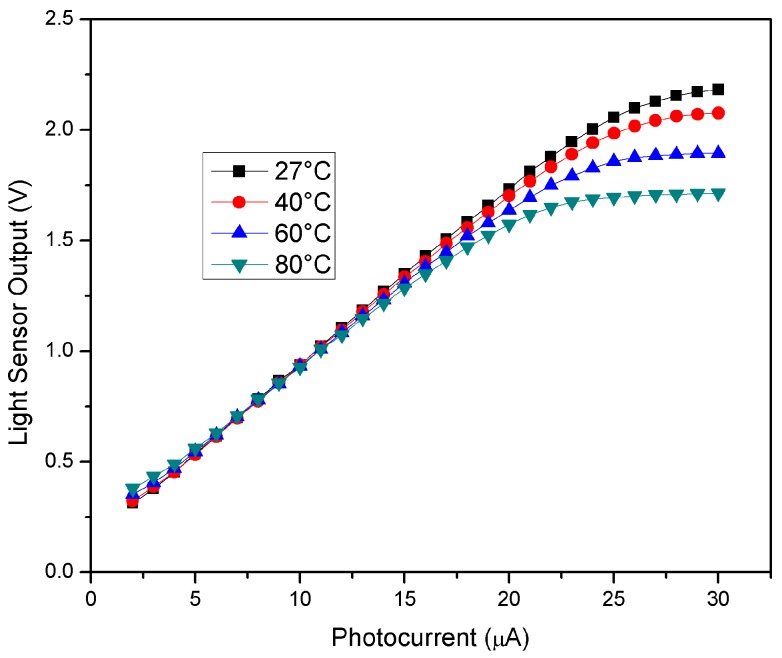
Light sensor output vs. photocurrent at different temperatures.

Temperature variations can affect the light sensor output. This dependence is shown in Figure 15, which demonstrates that, due to the amplifier non-idealities, the light reading saturates at lower and lower photocurrent values as the temperature increases.

### 4.3. RF Transmitter Performance

The RF performance of the implemented transmitter was characterized by analysing the transmitted signal received by a 0.4–3 GHz bandwidth, 5 dBi logarithmic periodic dipole antenna (model Rhode&Schwarz HL040) connected in turn to a Rhode&Schwarz FSV Spectrum Analyser. The antenna was placed at a distance of 1 m from the microchip.

**Figure 16:**
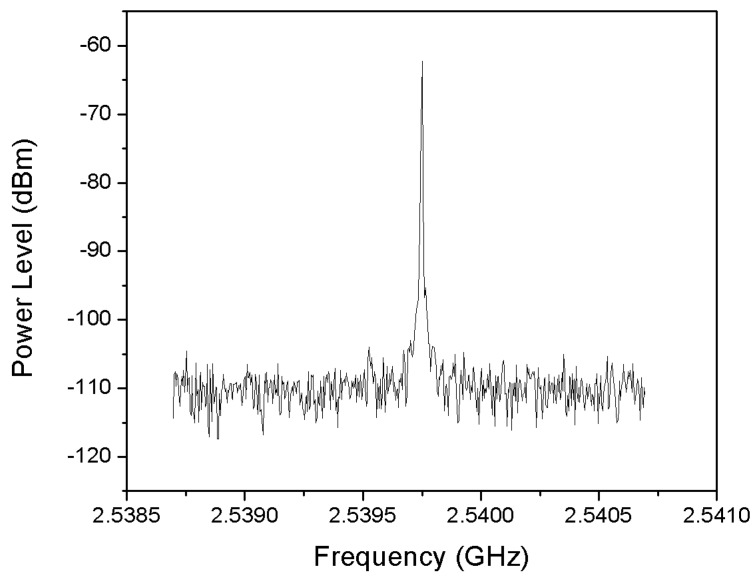
Frequency spectrum graph of the received signal.

The experimental measurements show a continuous wave (CW) signal, produced by the cross-coupled LC oscillator, at about 2.54 GHz as shown in Figure 16.

**Figure 17:**
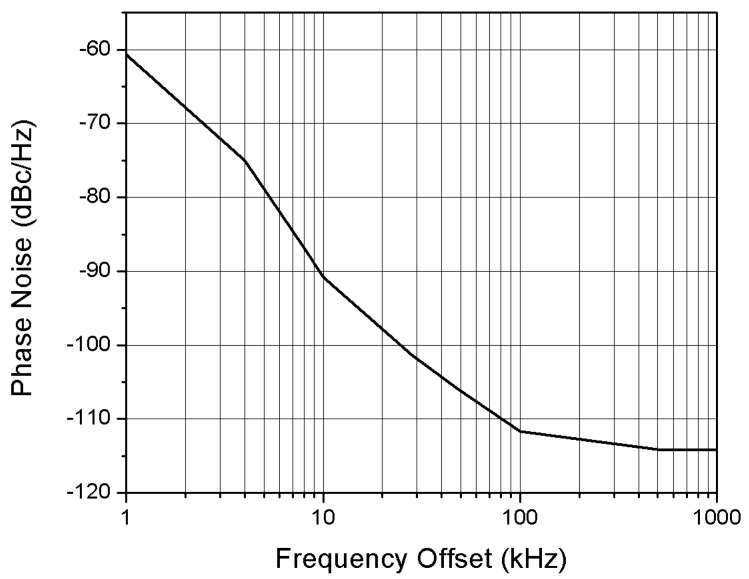
Phase noise of the received signal.

The proposed LC oscillator shows a phase noise of −90 dBc/Hz at 1MHz offset from the centre frequency, as shown in Figure 17. The phase noise is estimated as follows:

(11) $$L{\left(\Delta f\right)}_{dBc}=P_{s}\left(dBm\right)-P_{c}\left(dBm\right)-10log_{10}\left(\frac{RBW}{1\mathrm{Hz}}\right),$$

where $L{\left(\Delta f\right)}_{dBc}$ is the phase noise, $P_{s}$ is the noise power level in a band of 1 Hz at a certain offset frequency from the carrier central frequency *f*_0_, $P_{c}$ is the power level of the carrier at *f*_0_ and RBW is the bandwidth resolution of the spectrum analyser.

The LC transmitter shows a temperature sensitivity Δ*f*/Δ*T* = −0.25 MHz/°C.

### 4.4. Whole System Operation

The operation of the whole system was tested employing a commercial hand-held IC-R20 (Icom America Inc., 12,421 Willows Road NE, Kirkland, WA, USA) receiving unit placed at a distance of 1 m, although the transmitted RF signal was clearly detectable up to a distance of 1.5 m (Table 3). The assembled device was tested under the sun at a temperature of 24 °C and an irradiation level of 1125 W/m^2^. In agreement with the previous characterization, the output voltage of the PTAT and light sensor were 0.763 V and 1.971 V respectively.

**Figure 18:**
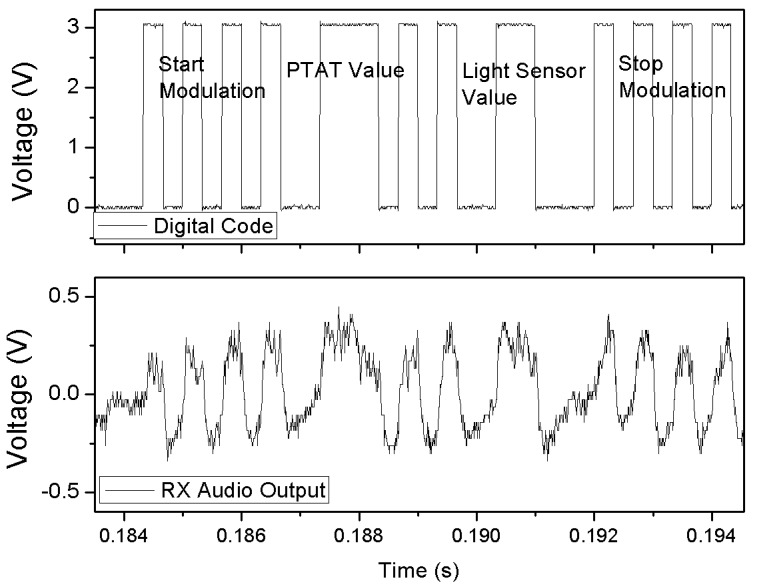
Sample transmitted code (**top**) and demodulated signal (**bottom**) as recorded at the output of the receiver. The bit stream contains the data produced by the PTAT and the light sensors.

The transmitted data bitstream is shown in Figure 18 (top) together with the recorded signal at the output of the radio receiver (bottom). Figure 18 (bottom) shows signal stability issues that could be suppressed by properly designing the AC-coupling of the demodulated signal to a low-frequency (audio) amplifier instead of that integrated in the used commercial receiver. The received values are 23.4 °C, 1118 W/m^2^.

## 5. Conclusions

In this paper, a full custom microchip realized in 0.35 μm CMOS technology, designed for ambient and processes monitoring, was proposed. It features a temperature and an illumination sensor. The temperature sensor features a sensitivity of 19.1 mV/°C and a linearity 0.99962 in the temperature range of 20 °C to 90 °C, while the illumination sensor, designed to operate in the range from 0 to 1200 W/m^2^, shows a sensitivity of 16.96 mV/W/m^2^ with a linearity of 0.99915. 

An integrated 2.5 GHz transmitter with an On-Chip-Antenna (OCA)-made in the top metal layer of the same die-transmits the collected data wirelessly by means of an on-off amplitude modulation scheme, at a bit rate of 3 kbit/s. The low bit rate was chosen in order to obtain that the decoded signal at the receiver output were in the audible band, as the receiver is a commercial low-cost radio receiving unit. However, simulations indicate that bit rates as high as 100 kbit/s could be easily obtained with the same technology.

## Figures and Tables

**Table 1:** Transistor dimensions of differential cross coupled LC oscillator.

	MP1	MP2	MN1	MN2	Mtail
**W**	72 μm	72 μm	72 μm	72 μm	100 μm
**L**	0.35 μm	0.35 μm	0.35 μm	0.35 μm	0.35 μm

**Table 2:** Performance summary of proposed PTAT sensor.

Power Supply (V)	Drawn Current (μA)	Area (mm^2^)	Temperature Range (°C)	Sensitivity (mV/°C)	Linearity
3.3	150	0.022	25–90	19.1	0.99962

**Table 3:** Comparison table with state-of-the-art sensors.

Ref.	Area (mm × mm)	Antenna Type	Reading Distance	Temperature Range (°C)	Additional Sensors
This work	1 × 4	Dipole	1.5 m	25–90	light
U. Calpa et al. [5]	1.5 × 1.5	Inductor	110 cm	15–35	no
Boram Kim et al. [22]	7.5 × 7.5	Inductor	5 mm	24–40	no
Arsalan et al. [8]	3.2 × 1.5	Spiral	3 cm	27–35	no
Jun Yin et al. [23]	0.9 × 1.25	External	4 m	−20–30	no
